# Prevalence and Correlates of Depression Among Pregnant Women Enrolled in a Maternal and Newborn Health Program in Rural Northern Ghana: a Cross-sectional Survey

**DOI:** 10.1007/s40609-020-00170-8

**Published:** 2020-05-07

**Authors:** Margaret Lillie, John A. Gallis, John Hembling, Raymond Kofi Owusu, Mohammed Ali, Safiyatu Abubakr-Bibilazu, Raymond Aborigo, Haliq Adam, Elena McEwan, John Koku Awoonor-Williams, Joy Noel Baumgartner

**Affiliations:** 1 Duke Global Health Institute, Durham, NC USA; 2 Duke Department of Biostatistics and Bioinformatics, Durham, NC USA; 3 Catholic Relief Services Head Quarters, Baltimore, MD USA; 4 Catholic Relief Services Country Office, Tamale, Ghana; 5 Navrongo Health Research Centre, Navrongo, Ghana; 6 Ghana Health Service, Accra, Ghana

**Keywords:** Antenatal, Depression, Ghana, Hunger, Hope, Maternal, Mental health

## Abstract

Women in many sub-Saharan African countries are at elevated risk of depression during pregnancy. However, there are still gaps in the estimates of antenatal depression and associated risk factors in very low-resource settings such as Northern Ghana. This study describes the prevalence of depression among rural pregnant women, participating in a maternal and child health program, in Ghana, and examines associated risk factors for depression. Pregnant women who were registered for group-based maternal and child health community programs were recruited for study participation from 32 communities in two rural districts in Northern Ghana (*n* = 374). Baseline surveys were conducted and depression was assessed using the Patient Health Questionnaire (PHQ-9). Bivariate and multivariable analyses used a modified Poisson and generalized estimating equations (GEE) model. Of the women in our study population, 19.7% reported symptoms indicative of moderate to severe depression (PHQ-9 score ≥ 10), with 14.1% endorsing suicidal ideation in the last 2 weeks. Bivariate analyses revealed that lower hopefulness, moderate and severe hunger, experiences of emotional, physical, and/or sexual intimate partner violence (IPV), and insufficient social support from female relatives were associated with symptoms indicating moderate to severe depression. In the multivariable analyses, low hopefulness, household hunger, emotional IPV, physical and/or sexual IPV, and insufficient female relative support remained significantly associated with depression. Antenatal depression is associated with unmet basic needs and safety. Perinatal mental health programming must take an ecological perspective and address personal, familial, and community-level factors.

## Introduction

### Background/Rationale

Women experience elevated rates of depression during the antenatal period in both high- and low-resource settings (Ayano et al. 2019; Fisher et al. 2012; Mahendran et al. 2019; Rahman et al. 2013; Sawyer et al. 2010; Weobong et al. 2014). The significance of addressing antenatal and maternal mental health is twofold in that both the mother and her child may experience the negative impacts. Understanding the risk factors for antenatal depression including comorbid life experiences is important in order to tailor prevention and treatment interventions during the antenatal period and subsequent parental stages. Global evidence indicates that poor mental health during the antenatal period is a risk factor for postnatal mental disorders (Josefsson et al. 2001; Robertson et al. 2004; Shamu et al. 2011; Weobong et al. 2014). Antenatal depression has been shown to be modestly linked to low birth weight infants, increased odds of premature delivery, decreased breastfeeding, excessive infant crying, infant diarrhea, affected infant sleep patterns, lower child cognition, and increased child antisocial behaviors (Bennett et al. 2016; Grigoriadis et al. 2013; Madlala and Kassier 2018; Smith et al. 2019; Stein et al. 2014; Wachs et al. 2009; Waters et al. 2014). Increasingly, global mental health researchers and policymakers are calling for a better understanding of social predictors of depression, such as access to resources and education, domestic violence, and social support, which may also affect child outcomes (Bennett et al. 2016; Collins et al. 2011; Rahman et al. 2013; Stein et al. 2014; Waters et al. 2014)*.* With the publication of World Health Organization’s Mental Health Global Action Plan (mhGAP), there are more tools for funders and programs to use when creating mental health programing for women in low-resource settings (WHO 2010; WHO 2016). However, without a better understanding of associated risk factors of perinatal mental health, we cannot effectively design and/or adapt mental health programs. While we know that mental distress exists among antenatal women in low- and middle-income settings, particularly those in the most socially and economically disadvantaged households, there is a lack of widespread local evidence to make informed programming decisions in specific contexts (Fisher et al. 2012).

Depression is the most commonly identified and studied mental disorder among pre- and postnatal women in Sub-Saharan Africa (Sawyer et al. 2010). Findings from a systematic review of determinants of antenatal depression in Ethiopia revealed that having low income, being unmarried, having debt, having experiences of food insecurity, and being between the ages of 20–29 years were associated with higher levels of antenatal depression (Ayano et al. 2019). A 2010 systematic review of mental health of perinatal women (antenatal and postnatal) in Sub-Saharan African (SSA) revealed some common psychosocial risk factors for perinatal depression including lack of social support, marital conflict, and being single (Sawyer et al. 2010). More recent studies in SSA have shown similar findings and revealed more details of factors associated with perinatal depression such as social support of female relatives being a strong protective factor for maternal mental health (Baumgartner et al. 2016; Garman et al. 2019). These more nuanced details such as types of social support, the role of household hunger, and experiences of intimate partner violence are needed to understand how to mitigate the onset of antenatal depression and/or tailor treatment within a certain social context (Baumgartner et al. 2016; Garcia et al. 2013; Golding 1999; Lutgendorf 2019; Maynard et al. 2018; Shamu et al. 2011; Sweetland et al. 2019). Additionally, while sociodemographic factors are most often measured in prevalence studies of depression, there is a gap in measurement of more internal factors such as hope, which may affect mental health (Le et al. 2015; Sikander et al. 2019). Hope has been studied as a correlate of lower levels of depression in high-income and middle-income countries but this has not been reviewed in detail in SSA or among antenatal populations more generally (Arnau et al. 2007; Griggs 2017; Kim and Shin 2014; Muyan and Chang 2016; Thimm et al. 2013; Thio and Elliott 2005).

Studies on perinatal mental health in SSA are becoming more common but there remain data gaps in lower resource SSA countries with much of the maternal mental health literature being clustered in Nigeria, South Africa, Uganda, and Ethiopia (Sawyer et al. 2010). While Ghana has transitioned to a middle-income country and is graduating from international health aid, there are still widespread economic, social, and health disparities between the urban south and the rural north of the country (Osei-Assibey 2014; Yamey et al. 2019). There are very few local studies or population-level studies assessing the prevalence and risk factors for antenatal mental health in Ghana. Existing literature includes a population-based study in rural Brong Ahafo Region which reported risk factors for antenatal depression including being in the lower wealth quintile, never been married, having had an unplanned pregnancy, and having had previous pregnancy complications (Weobong et al. 2014). Other literature on maternal depression in Ghana has focused on postnatal depression and depression of mothers with sick children (Gold et al. 2013; Ofori-Atta et al. 2019; Okronipa et al. 2012; Wemakor and Mensah 2016; Weobong et al. 2015).

### Objectives

The current study describes the prevalence of depression among rural pregnant women in northern Ghana who are participating in a community-based, maternal and child health promotion interventions and examines associated risk factors for depression in women who are pregnant. This study adds to the dearth of literature on antenatal depression in rural Ghana. Increasingly, the governments in low-resource settings are looking to implement broad, community-level maternal mental health programs; however, these programs could be too resource intensive and may benefit from more targeted interventions if there was more evidence to target those at higher risk. Not only will this study add to the existing literature on known correlates of depression like IPV and indicators of poverty in SSA but it will also explore less-understood correlates like self-reported hope and social support. These lesser understood measures could have implications for understanding other important components and measures of quality mental health programming.

## Methods

### Parent Study

We examined pre-intervention baseline data obtained from a longitudinal, cluster randomized control trial (cRCT) (NCT03665246) that is evaluating the impact of the Integrated Mothers and Babies Course (iMBC) (Le et al. 2015; Le et al. 2010) being administered by Catholic Relief Services (CRS), an international, non-governmental organization (NGO) working in Northern Ghana in collaboration with the Ghana Health Service. Each community group was randomized to participate in one of two maternal, newborn and child health (MNCH) programs—one that included maternal mental health content (iMBC) and one that did not (Community Pregnancy Surveillance and Targeted Education Sessions [C-PrES]). Both programs are implemented by CRS via the Rural Emergency Health Service and Transport project (REST II). The baseline questionnaire was completed between August 27 and September 6, 2018, before the participants started attending the program for which their community had been randomly assigned. We targeted recruitment of 16 clusters per arm (32 total clusters). We assumed a standardized effect size on the primary trial outcome of approximately half a standard deviation (i.e., 0.5), and an ICC of 0.04 based on preliminary data from a similar study. Allowing for the possibility of one cluster dropping out, we have 81.8% power to detect this effect size at an alpha of 0.05, with an average of 7 total participants per group and a large coefficient of variation of cluster sizes of 0.850. The average cluster size ended up being over 11 per group, leading to a final baseline sample size of 374 and > 90% power. This manuscript adheres to the STROBE Statement guidelines for reporting cross-sectional studies (von Elm et al. 2007).

### Setting

The study was conducted in 32 communities (clusters) in two districts in Northern Ghana: West Mamprusi District found in the North East Region and Nabdam District found in the Upper East Region. Each district has a unique language; Mampruli is spoken in West Mamprusi and Nabt is spoken in Nabdam District. Each community had one available women’s group in which all pregnant women in the community could enroll, resulting in 32 women’s groups across the study participants.

### Study Population

Participants were recruited to the study because they had been enrolled in CRS-administered (MNCH) programs (iMBC or C-PrES). To participate in the study, the women needed to be pregnant at the time of the survey, 16 years of age or older, be enrolled in the MNCH health programs, and plan to maintain residence in the community for the duration of the program. All participants signed a written informed consent form, or, if they were illiterate, were read the consent form and provided their fingerprint with a witness signature.

### Survey Translation

Because Mampruli and Nabt are not commonly written languages, the survey was in English and was translated at the time of each interview by the local enumerators. Before conducting the survey with participants, the enumerators participated in a week-long training in which they reviewed the survey content, practiced the survey administration in English, and then practiced the survey administration in their local language with support from Duke University and CRS leadership. Because the PHQ-9 was the main outcome measure, the PHQ-9 items were forward translated into Mampruli and Nabt and then back translated into English. Translators had a college degree, were from the two implementation districts, and participated in both the programmatic and research trainings. Items that did not translate properly were discussed between the leadership and the local translators to create a final, translated scale. The entire survey, including the PHQ-9, was pretested in the field with local mothers, outside the study communities, who were participating in other CRS programming.

### Data Collection

Data was collected on Samsung Galaxy tablets using CommCare, a HIPAA (The United States Health Insurance Portability and Accountability Act of 1996) compliant, cloud-based, and field data collection and reporting platform, published by Dimagi, Inc. Each participant was interviewed by a local enumerator in her local language (Mampruli or Nabt) and in a private location in her community. Data collectors were trained to offer referrals to participants who endorsed self-harm/suicidal ideation within the mental health scale items and/or who reported physical or sexual intimate partner violence (IPV) to the appropriate community or district level support per guidance from the Ghana Health Service.

### Outcome Measure

For our primary mental health measure, we used the Patient Health Questionnaire (PHQ-9), a common depression screener that has been previously validated and recommended for use in Ghana (though, not in the Mampruli or Nabt languages), to assess depression symptoms (Kroenke and Spitzer 2002; Kroenke et al. 2001; Weobong et al. 2009). The nine items of the PHQ-9 are summed for a score between 0 and 27. The PHQ-9 has standard categorizations: minimal or no depression (score of 0–4), mild depression (score of 5–9), moderate depression (score of 10–14), moderately severe depression (score of 15–19), and severe (score of 20–27) (Kroenke and Spitzer 2002; Kroenke et al. 2001). For analysis, we dichotomized the PHQ-9 variable into those who would have screened for no treatment follow-up in a clinical setting (none or mild depression (score of less than 10)) and those who would have screened for a treatment plan in a clinical setting (moderate to extremely severe depression (score of 10 or greater)) (Kroenke and Spitzer 2002). The PHQ-9 scale in our study population had strong internal consistency (*α* = 0.81). To report self-harm, we dichotomized the suicidality item from the PHQ-9 into no (did not report thoughts of self-harm in the past 2 weeks) and yes (did report thoughts of self-harm in the past 2 weeks by responding several days, more than half the days, or nearly every day).

### Correlates

With an eye towards better understanding internal factors associated with depression, we included the Herth Hope Index to assess hope, a 12-item scale in which respondents state their agreement or disagreement with each item. Items include statements such as “I have a positive outlook towards life,” “I have short and long range goals,” and “I can see possibilities in the midst of difficulties,” with some items being reverse coded (e.g., “I feel all alone”). The scores for each item are added up to create a continuous score from 0 to 48 with a higher score indicating a higher level of hope (Herth 1991).

We also used the Household Hunger Scale (HHS), created by the USAID funded Food and Nutrition Technical Assistance program (FANTA III), to assess food insecurity. HHS is a six-question scale (three topical questions [no food in the house, hunger when going to sleep, lack of food in 24 h] which are each followed by questions asking the frequency of occurrence) which is scored to create a score from 0 to 6 with higher scores indicating higher levels of food insecurity. We used the standard categorical variable indicating little to no hunger (0–1 point), moderate to severe hunger (2–3 points), and severe hunger (4–6 points) (Ballard et al. 2011).

Marital control and IPV during the past 12 months were assessed using standard items from the 2008 Ghana Demographic and Health Survey (DHS) (40). Those who had a partner in the last 12 months were asked the martial control and the IPV questions. Domains were specified using the *Guide to DHS* Statistics (controlling behaviors, emotional IPV, physical IPV, sexual IPV) (Croft et al. 2018; Ghana Statistical Service et al. 2009). For analysis, each domain was represented using a binary variable indicating if the participant endorsed at least one item within that domain. Physical and sexual IPV were collapsed into one domain for analysis because these were the IPV domains that required a referral during data collection for our study population.

To measure socioeconomic status (SES), we used the Ghana Equity Tool (all items listed in Table 1), which uses data from the DHS to create a list of assets that may be important indicators of household wealth (Ghana Equity Tool 2016). We added two additional questions about assets that the local data collection team in Ghana thought were important assets that may determine wealth (Does your household have a satellite dish? Does anyone in your household use mobile money?). To make one single SES variable, we generated an asset index using the polychoric correlation principal component analysis approach, as described by Kolenikov and Angeles and Maselko et al., to create five wealth quintiles based on the Ghana Equity Tool and the 2 additional questions listed above (Kolenikov and Angeles 2009; Maselko et al. 2018). Those items in the equity tool that did not show much variability (> 90% owned or did not own asset) were excluded from the creation of the asset index.

**Table 1:** Sociodemographic characteristics and bivariate associations between depression status and covariates adjusted for clustering by community

Variable	Total *N* = 374	None/mild depression *N* = 300	Moderate/moderately severe depression *N* = 74	Risk ratio (95% CI)	*p* value
Mean Hope Score (SD) (min, max)	38.1 (3.8) (27, 48)	38.5 (3.8) (30, 48)	36.8 (3.5) (27, 46)	0.91 (0.86, 0.96)	0.002
Mean age (SD) (min, max)	27.0 (6.8) (16, 50)	27.0 (6.9) (16, 50)	26.8 (6.4) (17, 40)	0.99 (0.97, 1.02)	0.628
Age					0.509
≤ 24	40.4%	41.0%	37.8%	REF	
25–34	41.2%	39.7%	47.3%	1.15 (0.82, 1.63)	
≥ 35	18.4%	19.3%	14.9%	0.86 (0.49, 1.50)	
Mean # of pregnancies (SD) (min, max)	3.3 (1.9) (1, 9)	3.4(2.0) (1, 9)	3.2 (1.8) (1, 7)	0.96 (0.87, 1.06)	0.460
# of pregnancies					0.758
One pregnancy	21.9%	21.0%	25.7%	REF	
2–3 pregnancies	34.0%	34.7%	31.1%	0.82 (0.47, 1.45)	
4 or more pregnancies	44.1%	44.3%	43.2%	0.85 (0.51, 1.42)	
Ever attended formal school	51.3%	50.7%	54.1%	1.03 (0.74, 1.44)	0.860
Hunger					< 0.001
Little to none	73.0%	78.0%	52.7%	REF	
Moderate	25.1%	20.7%	43.3%	2.45 (1.68, 3.58)	
Severe	1.9%	1.3%	4.1%	2.76 (1.10, 6.90)	
Assets in household per Ghana Equity Tool:					
Color TV^a^	45.7%	46.3%	43.2%	--	--
Agricultural land	97.3%	98.0%	94.6%	--	--
Refrigerator	8.6%	9.0%	6.8%	--	--
Video deck/DVD^a^	36.4%	36.3%	36.5%	--	--
Bank account^a^	23.9%	25.4%	17.6%	--	--
Electricity^a^	63.6%	62.7%	67.6%	--	--
Clock^a^	84.8%	84.0%	87.8%	--	--
Cupboard	3.5%	4.0%	1.4%	--	--
Fuel (wood)	96.2%	96.0%	97.3%	--	--
Toilet (no facility/bush) ^a^	52.1%	49.7%	62.2%	--	--
Non-sachet drinking water	100%	100%	100%	--	--
Floors (cement) ^a^	87.2%	86.0%	91.9%	--	--
Exterior walls (cement) ^a^	15.0%	16.0%	10.8%	--	--
Mobile money (non-Equity Tool item) ^a^	69.0%	70.5%	63.0%	--	--
Satellite dish (non-Equity Tool item) ^a^	22.1%	22.1%	21.9%	--	--
SES (asset quintile)					0.969
Lowest quintile	19.7%	19.9%	19.2%	REF	
Second quintile	19.7%	19.5%	20.6%	1.01 (0.62, 1.64)	
Middle quintile	20.5%	20.5%	20.6%	1.00 (0.59, 1.70)	
Fourth quintile	18.6%	18.2%	20.6%	0.95 (0.62, 1.46)	
Highest quintile	21.4%	21.9%	19.2%	0.77 (0.35, 1.66)	
Living with partner	89.8%	88.7%	94.6%	2.34 (0.86, 6.41)	0.097
Currently in a polygynous relationship^b^	39.7%	38.1%	46.5%	1.32 (0.91, 1.92)	0.147
Physical health					0.065
Fair/poor	15.5%	13.3%	24.3%	REF	
Good	36.1%	36.3%	35.1%	0.63 (0.39, 1.03)	
Very good/excellent	48.4%	50.3%	40.5%	0.59 (0.38, 0.94)	
Controlling behaviors (12 months)^c^	79.1%	77.7%	84.5%	1.47 (0.76, 2.85)	0.251
Emotional IPV (12 months)^d^	44.6%	39.9%	63.4%	1.96 (1.35, 2.87)	< 0.001
Physical and/or sexual IPV (12 months)^e^	38.5%	33.8%	57.7%	1.93 (1.25, 2.98)	0.003
Reported thoughts of self-harm or suicide in past 2 weeks (item in PHQ)	14.2%	9.7%	32.4%	2.81 (1.78, 4.46)	< 0.001
Sufficient social support					
Husband^f^	45.8%	48.2%	36.5%	0.78 (0.54, 1.11)	0.177
Male relatives	22.7%	24.7%	14.9%	0.61 (0.32, 1.16)	0.131
Female relatives	36.9%	40.7%	21.6%	0.52 (0.30, 0.90)	0.012
Female friends	17.4%	18.7%	12.2%	0.70 (0.36, 1.35)	0.291

^a^Included in the asset index

^b^Out of *n* = 360 (*n* = 298 none to mild; *n* = 73 moderate to moderately severe) who reported being married

^c^*n* = 354 (total); *n* = 283 (none to mild); *n* = 71 (moderate to moderately severe)

^d^*n* = 359 (total); *n* = 288 (none to mild); *n* = 71 (moderate to moderately severe)

^e^*n* = 358 (total); *n* = 287 (none to mild); *n* = 71 (moderate to moderately severe)

^f^*n* = 371 (total); *n* = 291 (none to mild); *n* = 74 (moderate to moderately severe)

To measure social support, we asked participants 4 separate questions about social support received from male relatives, female relatives (including co-wives, sisters, sister-in-laws, aunts, and nieces), female friends, and their husband or partner. Participants reported if the support they received from these groups was sufficient, insufficient, or if they never received support from each type of relationship. These variables were dichotomized into sufficient and insufficient (insufficient and never).

Additional questions included relationship status, whether they had ever attended formal education, and self-reported physical health.

### Statistical Methods

Bivariate and multivariable analyses used a generalized estimating equation (GEE)–modified Poisson model, with an exchangeable working correlation to account for clustering by community. The modified Poisson model allows us to obtain risk ratios, which are more readily interpretable than odds ratios (Gallis and Turner 2019; Zou and Donner 2013). We first examined the relationship between each predictor and depression status. The final models for the multivariable analyses included a priori variables that are theoretically known risk factors for depression (hope, IPV, hunger, number of pregnancies, education, and SES) and also emergent predictor variables that were significant in the multivariable analyses (*p* < .05). Because emotional IPV and physical and/or sexual IPV are highly correlated (correlation of 0.48 in our study sample), two multivariable models were run, one that included emotional IPV and not physical and/or sexual IPV and one that included physical and/or sexual IPV and not emotional IPV.

While our original study was not powered for comparison across districts, given the much smaller number of clusters and participants within each district, exploratory, post hoc, multivariable analyses were run stratified by district.

## Results

All pregnant women who were participating in either the C-PrES or iMBC groups agreed to participate in the study. No one refused to participate in the study who was approached. At baseline, 378 women registered for the research and completed the baseline survey. However, for the final dataset and analyses, this was revised to 374 women as we discovered at the follow-up survey timepoints that four women had completed the baseline survey twice with separate research assistants (participants received soap as a gift and may have taken the survey again to receive more soap). The survey that was completed first was kept in the data set. All 374 participants completed the PHQ-9. The mean PHQ-9 score was 6.3 (standard deviation [SD] = 4.1), with a range of 0 to 16. No participants scored in the severe range of the scale (20–27).

Sociodemographic and health characteristics for the study participants enrolled at baseline are shown in Table 1. The mean age of the research participants was 27 years (SD = 6.8) with an average of about three lifetime pregnancies (SD = 1.9). Ninety percent of the study population was living with a partner, with about 40% overall in a polygamous marriage. About half of the study population had never received any formal education and 27% were experiencing moderate or severe hunger in their households. About one-fifth of the population screened for moderate to moderately severe depression (37.2% none/minimal depression; 43.1% mild depression; 16.6% moderate depression; 3.2% moderately severe depression) and 14.2% reported thoughts of self-harm or suicide in the last 2 weeks. In West Mamprusi, 17.9% of participants reported symptoms of moderate to moderately severe depression and in Nabdam, 25.4% of participants reported symptoms of moderate to moderately severe depression (no statistically significant difference). Rates of controlling behaviors and IPV (in the last 12 months) were high with 79.1% reporting controlling behaviors, 44.6% reporting emotional IPV, and 38.5% report physical and/or sexual IPV. About 40% of our study population fell within the two lowest wealth quintiles per the Ghana Equity Tool estimates.

Bivariate analyses of study population characteristics by mental health status (none/mild depression and moderate/severe depression) are illustrated in Table 1. For the bivariate analyses, we tested associations for some items as both categorical and continuous variables. Results indicate that those with moderate (risk ratio (RR = 2.45, 95% confidence interval (95% CI) (1.68, 3.58)) to severe (RR = 2.76, 95% CI (1.10, 6.90)) hunger were more likely to indicate symptoms of moderate to moderately severe depression. In addition, those who experienced emotional (RR = 1.96, 95% CI (1.35, 2.87)) or physical and/or sexual (RR = 1.93, 95% CI (1.25, 2.98)) IPV in the past year and those who report thoughts of self-harm or suicide in the past 2 weeks (RR = 2.81, 95% CI (1.78, 4.46)) were more likely to experience symptoms of moderate to moderately severe depression, while those who indicated sufficient social support from female friends (RR = 0.52, 95% CI (0.30, 0.90)) and higher levels of hope (RR = 0.91, 95% CI (0.86, 0.96)) were less likely to indicate symptoms of moderate to moderately severe depression.

Results of the two multivariable analyses are reported in Table 2. The multivariable analysis included hope, emotional IPV or physical/sexual IPV, hunger, number of pregnancies, education, SES, and social support from female relatives. Women’s age was not included because it is highly correlated with number of pregnancies and self- harm was not included because it is an item from the PHQ-9 outcome measure. Of the 374 participants, 15 were missing emotional IPV data and 16 were missing physical and/or sexual IPV data, so only 359 and 358 participants were included in the final multivariable models, respectively. In the multivariable analysis, low hopefulness, household hunger, emotional and physical and/or sexual IPV, and insufficient support by female relatives remained significantly associated with moderate to moderately severe depression. Most notably, those with severe hunger and those with moderate hunger had over two times the risk of having moderate to moderately severe depression than those with little to no hunger.

**Table 2:** Multivariable associations among pregnant women between depression status and covariates adjusted for clustering by community

	Multivariable model including emotional IPV, excluding sexual/physical IPV^a^		Multivariable model including sexual/physical IPV excluding emotional IPV^b^	
Predictor	Risk ratio (95% CI)	*p* value	Risk ratio (95% CI)	*p* value
Hope Score	0.92 (0.87, 0.98)	0.006	0.93 (0.87, 0.98)	0.012
Number of pregnancies		0.428		0.477
One pregnancy	REF		REF	
2–3 pregnancies	0.74 (0.43, 1.29)		0.76 (0.43, 1.32)	
4 or more pregnancies	0.69 (0.38, 1.24)		0.71 (0.39, 1.27)	
Ever attended formal school	1.00 (0.66, 1.51)	0.993	0.99 (0.65, 1.54)	0.991
Household hunger		< 0.001		< 0.001
Little to none	REF		REF	
Moderate	2.20 (1.47, 3.29)		2.23 (1.48, 3.37)	
Severe	2.66 (1.02, 7.01)		2.90 (1.15, 7.32)	
SES (asset quintile)		0.297		0.487
Lowest quintile	REF		REF	
Second quintile	1.36 (0.86, 2.16)		1.38 (0.87, 2.20)	
Middle quintile	1.24 (0.76, 2.04)		1.27 (0.76, 2.13)	
Fourth quintile	1.48 (0.90, 2.44)		1.38 (0.84, 2.27)	
Highest quintile	1.22 (0.52, 2.85)		1.25 (0.55, 2.84)	
Emotional IPV (12 months)	1.56 (1.06, 2.28)	0.023	-	-
Physical and/or sexual IPV (12 months)	-	-	1.54 (1.04, 2.27)	0.032
Sufficient social support from female relatives	0.58 (0.34, 1.00)	0.052	0.57 (0.34, 0.95)	0.029

Inclusive of hope, emotional IPV or physical/sexual IPV, hunger, number of pregnancies, education, SES, and social support from female relatives

^a^*n* = 359

^b^*n* = 358

When stratified by district, the multivariable models, while still consistent with the combined multivariable models, show stronger associations in Nabdam District between moderate to moderately severe depression and many of the predictors, including emotional IPV and physical and/or sexual IPV. The association between moderate or severe hunger and moderate to moderately severe depression was strong in both districts [data not shown].

## Discussion

Our results indicate that 19.7% of our study population have moderate to moderately severe depression during pregnancy, confirming the need to address depression during the antenatal period. Moreover, even though none of the participants was categorized into the severe depression category, 14% of all the participants reported thoughts of self-harm or suicidal ideations, highlighting the severity of mental health symptoms experienced by some during pregnancy. Antenatal depression will not be improved, however, if basic needs and safety are not being met. Most notably, we found that household hunger is strongly associated with antenatal depression.

While previous studies in SSA and Ghana have investigated an asset-based SES index in relation to antenatal and perinatal depression, this is the first study in Ghana that investigated the association between household hunger specifically and antenatal depression (Sawyer et al. 2010; Wemakor and Mensah 2016; Weobong et al. 2014; Weobong et al. 2015). While SES may be considered a predictor, household hunger status in relation to depression gives a more immediate picture of the current context in which these women are living.

While we found that those with higher levels of hope had a lower risk of indicating symptoms for moderate to moderately severe depression, hope was generally very high in this population with the scores being heavily skewed towards higher scores. Given that this population is reporting higher levels of hope, mental health programmers should explore how to build upon this strong, internal asset to engage their program populations. Further research should include measures of hope to confirm our findings and to better understand how hope may be related to depression symptoms.

Emotional IPV, physical and/or sexual IPV, and social support from female relatives remain significant in the multivariable models, which is consistent with the literature on correlates of depression in SSA and Ghana (Sawyer et al. 2010; Weobong et al. 2014; Weobong et al. 2015). Given these findings, IPV and social support cannot be considered without also considering how they are related to other factors such as household hunger or other socioeconomic measures. Additionally, mental health programs should ensure that they are appropriately referring women who are experiencing IPV to locally available resources and/or properly engaging with men to decrease the perpetration of violence. While our finding that social support is correlated with depression status is not new, our study was able to provide a more nuanced view of social support from whom and the potential impact of social support from different types of relationships. Future studies should examine in more depth the role that female relatives as well as other social support systems play in the lives of pregnant women.

This study has a number of strengths and limitations. Due to the cross-sectional nature of our study, we are unable to determine directionality of the presented associations. While we assume one-way causality based on our theoretical frameworks (e.g., household hunger causes depression), causality could be opposite (e.g., depression causes household hunger perhaps due to decreased economic activity) or be a two-way causality. The study only collected data in two rural districts in northern Ghana and therefore is not generalizable to the general pregnant Ghanaian population. However, a strength is that the scope of data collection across 32 communities lends important data for mental health programming in pregnant populations in much of Northern Ghana which has similar socioeconomic and demographic characteristics. While the maternal and child health program from which we sampled was offered to all pregnant women in the study communities, we do not know if we captured the most vulnerable women who did not have the time or resources to participate in the program or in the study. Some of our measures such as social support and IPV allowed us to take a more nuanced look at the social predictors of antenatal depression while others, such as the Equity Tool, may have limited our scope. The equity tool in particular did not have variability for pertinent asset variables such as water sources, fuel sources, and the owning of agricultural land.

## Implications for Policy and Practice

Mental health programming needs to be cognizant of how to ensure the basic needs of pregnant women are being met and how they can better connect these women to resources or empower women to become more resilient to daily stressors. Given our results that hunger and poor mental health are associated, authorities conducting mental health programming should explore opportunities for integration with food security programs to support household access to food, particularly during lean seasons in which food may be scarce. Additionally, given the relationship between IPV and social support with depression status, mental health programming could engage women at a community level and directly address their relationships with each other and with their nuclear and extended families.

## Conclusion

While improving women’s mental health during the antenatal period is important for the outcomes of mothers and their children, perinatal mental health programming may not realize its full potential to be impactful if programs fail to address familial, personal, and community-level factors, such as household hunger and IPV.

## Data Availability

The dataset generated and/or analyzed during the current study is not publicly available but is available from the corresponding author on reasonable request.
